# Pneumorrhachis and hyponatremia after a neck hack—A case report

**DOI:** 10.1016/j.ijscr.2020.02.063

**Published:** 2020-03-03

**Authors:** Tommy Supit, Ajid Risdianto, Dody Priambada, Muhamad Thohar Arifin, Happy Kurnia Brotoarianto

**Affiliations:** a Department of General Surgery, Faculty of Medicine, Diponegoro University, Semarang, Indonesia; b Department of Neurosurgery, Neurospine Division, Faculty of Medicine, Diponegoro University, Semarang, Indonesia; c Department of Neurosurgery, Faculty of Medicine, Diponegoro University, Semarang, Indonesia

**Keywords:** Pneumorrhachis, Pneumocele, Spinal cord injury, Cervical, Penetrating

## Abstract

**Introduction:**

Penetrating cervical spinal cord injury (SCI) is a rare clinical entity that requires a multitude of health care specialists for proper management. The unpredictable nature of penetrating SCI and complex systemic sequela contribute to the high mortality rates of penetrating SCI.

**Presentation of case:**

An 18-year-old-male patient was admitted to the emergency department with tetraparesis following a penetrating injury to the neck. Radiological examination revealed fractures of C4 and C5 spinous processes and extensive intradural pneumorrhachis. The patient was managed operatively with laminectomy, vertebral augmentation, and duroplasty. An acute decreased level of consciousness was observed four days after the operation. Laboratory investigation revealed critically low plasma sodium level. The patient remained decerebrated despite electrolyte correction and pronounced brain dead on the seventh postoperative day.

**Discussion:**

Metabolic derangements and pulmonary physiologic changes following trauma are lethal complications. Hyponatremic encephalopathy and disrupted pulmonary function caused by high cervical compression by the extensive pneumorrhachis contributes to the morality in this case report.

**Conclusion:**

This case report presents a rare clinical entity along with its’ complications. Prompt clinical stabilization, strict biochemical monitoring, and multidisciplinary care from health care specialists are mandatory for SCI patients.

## Highlights

• A rare case of pneumorrhachis after a cervical penetrating injury.
• Presentation of metabolic, cardiopulmonary derangements and other biochemical sequela of penetrating cervical injury.
• The importance of multidisciplinary team effort for the management of penetrating cervical injury.

## 1 Introduction

Penetrating cervical spinal cord injury (SCI) is rarely encountered and is a challenge even for experienced clinicians [1]. The heterogeneous nature of the injury, risk of infection, spinal shock, and other metabolic derangements makes it less predictable than the majority of spinal trauma cases. An even rarer clinical entity is pneumorrhachis, which currently has no established treatment guideline [2]. This manuscript reports on a case of an 18-year-old man who suffered from a penetrating injury to the posterior neck that developed into a large pneumorrhachis and its biochemical sequela. This work is reported by following the surgical case report (SCARE) guideline [3]. Ethical approval exempted by the hospital’s board of ethics.

## 2 Presentation of case

**Fig. 1:**
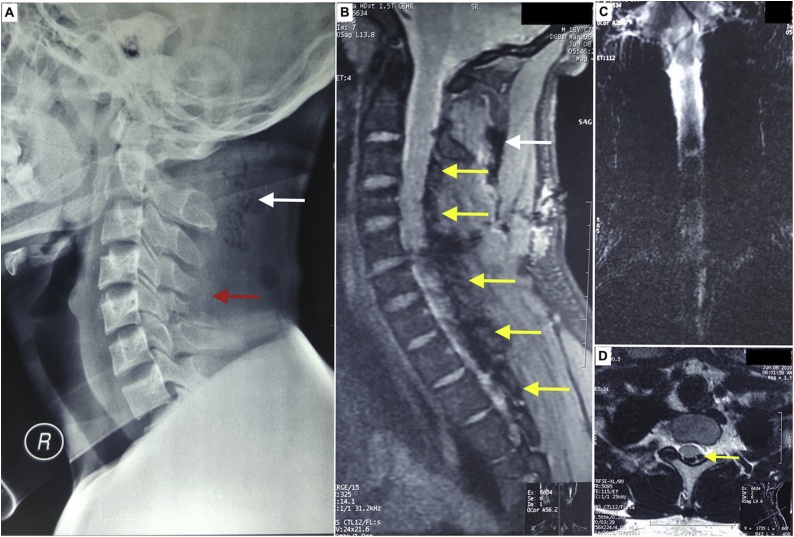
Radiological examinations **A**: Lateral cervical x-ray showing fractures of C4 and C5 spinous process (red arrow) and subcutaneous emphysema (white arrow). **B**: Sagital T1 cervical MRI showing subcutaneous emphysema anterior to the splenous capitis muscle (white arrow), disruption of soft tissue along the path of the blade that transected the spinal cord between C5-C6 and pneumorrhachis extending from C2–T1 (yellow arrows). **C**: Coronal T2 cervical MRI showing impaired cerebrospinal fluid flow caudal to the level of C2. **D**: Axial T1 cervical spine showing compression of the spinal cord due to trapped air (yellow arrow) within the spinal canal.

An 18-year-old-male was presented with tetraparesis after a penetrating wound to the cervical spine. Twelve hours before admission the patient was a victim of a hit-and-run while riding on the back of a motorcycle when an assailant hacked his neck with a blade while driving from the opposite direction. The weapon used was described by the patient to be a modified machete-like blade with the tip curved forward. The patient managed to get off his motorcycle in an attempt to pursue the attacker, whoever he fell after a few steps and unable to get up after experiencing weaknesses on all four extremities. He suffered from a transverse penetrating skin wound approximately 3 cm in length to the neck. The patient received simple wound closure in the primary health care center before being transferred to our hospital. Upon primary survey, the patient was found to be fully alert with stable vital signs. Physical examination showed tetraparesis with significantly weaker lower extremities with a preserved soft touch and pinprick sensation above the C6 dermatome. There was no sensory or motor function in the sacral segments (ASIA-A). Radiographic examinations revealed soft tissue disruptions along the injury path, subcutaneous emphysema, fracture of C4–C5 spinous processes, intradural pneumorrhachis extending from C2–T1 and partial transection of the spinal cord at the level of C5–C6 (Fig. 1).

**Fig. 2:**
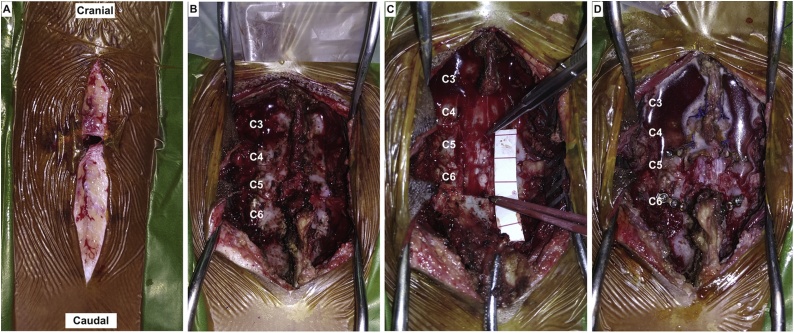
Intraoperative findings **A**: Tranverse penetrating wound measuring 1 cm. Considerable contamination of the subcuntaneous tissue was observed. **B**: The fractured spinous processes of C4–C5 and transverse C5-C6 intervertebral entrance wound. **C**: Laminectomy of C4–C6 revealed a 3 cm linear longitudinal disruption of the spinal dura mater. **D**: Post duroplasty, laminoplasty and full-augmentation of C4 and C6 with mini-plates.

The patient was initially managed with ATLS principles along with a broad-spectrum antibiotic before being transferred to the neurology ward. After four days of clinical stabilization, the patient showed no neurological improvement and subsequently underwent debridement, laminectomy, duroplasty and vertebral augmentation of the C4–C6 (Fig. 2). The patient regained full consciousness after the anesthesia and underwent a relatively well post-operative recovery within the first 3 days while showing an equivocal neurological function. An acute decreased level of consciousness was observed 4 days post-operation requiring mechanical ventilation. Laboratory investigation revealed a plasma sodium level of 114 mmol/L. Prompt electrolyte correction with conservative measures for cerebral edema was initiated. However, the patient remained decerebrated and pronounced brain dead 7 days post-operation. Cardiorespiratory failure that results from hyponatremic encephalopathy is determined to be the cause of death of this case.

## 3 Discussion

Penetrating spinal injury can be categorized into missile penetrating (MPSCI) and a non-missile penetrating spinal injury (NMPSCI) such as a stab wound. A cervical stab wound is a rare clinical entity that constituted approximately only 1% of all SCI [1]. Several case reports described the rare incidence of NMPSCI, where it is more prevalent in areas of military conflicts and predominantly affects males [4]. In most cases, the victims are usually stabbed once, and the attacker usually withdraws the object form the victim’s body; like in this case report. Neurological deficit ranges from asymptomatic dural tears, neuropraxia to complete spinal cord injury. The most common incomplete spinal cord injury that results from NMPSCI is the Brown-Sequard syndrome, which results in better prognosis than the other type of incomplete SCI [5,6].

The heterogeneous and unpredictable nature of the injury of NMPSCI complicates its management, as illustrated in this case. The axis of the laceration on the level of cutaneous tissue (transverse) and spinal cord (longitudinal) was perpendicular to each other. Such unexpected findings might be attributed to the bizarre shape of the weapon and the traumatology. The transverse orientation of the entrance wound and its diagonal tract through the cervical tissue is consistent with a hacking movement of the assailant from the victim’s side. The forward-tipped curve of the blade might have entered the neck transversely, lodged in the cervical vertebra and then twisted longitudinally as the assailant drove away creating a hollow subcutaneous wound and longitudinal spinal cord laceration. The fractured spinous processes demonstrate the heaviness of the blade and the relatively high-energy force. The air entered the spinal canal directly through the damaged spinal tissue.

Pneumorrhachis (PR), synonymous with spinal pneumocele, epidural pneumotosis or emphysema denotes the presence of intraspinal air [[7], [8], [9]]. The etiology of PR can be classified into iatrogenic, traumatic and nontraumatic. Several cases reports described conditions that may directly or indirectly produce PR such as barotraumas, physical exertions, respiratory complications, malignancy and its associated therapy [[10], [11], [12]]. In this traumatic case, the air entered the intraspinal space through the gaping neck wound and entrapped to a one-way air valve mechanism created by the paraspinal soft tissues. The management of PR is individualized according to its etiology with the majority of cases to be asymptomatic and regresses spontaneously with air resorption into the vascular system. Thus, PR is usually treated conservatively. Although the use of prophylactic antibiotics for PR is still debated [2], we considered the nature of the injury and the degree of contamination of this case mandates its use to prevent meningitis. Operative treatment for ASIA A cord injury yields poor neurological results [13]. The rationale for operating on this patient was decompression that might have cause ongoing spinal shock, debridement, and spinal stabilization to allow early rehabilitation. However, the neurological symptoms were undetermined whether it is caused by the transection of the spinal cord, spinal shock or PR.

Another problem that complicates the management of this patient was hyponatremia. This metabolic derangement is frequently encountered after traumatic cervical SCI [14]. A retrospective study involving 33 patients with acute spinal trauma described the rate of hyponatremia to be as high as 85.7%, which was more frequent in patients with SCI [15]. Hyponatremia persisted longer (24 h – 12 days vs. 2–3 days) and in greater degree (131.7 ± 0.7 vs. 136.7 ± 1.3 mmol/L) in SCI compared to the non-SCI group. The study suggests that hyponatremia is associated with the intact renin-angiotensin system responding to neural injury. Six additional theories that explain the pathogenesis of hyponatremia following traumatic SCI [1]: loss of supraspinal control of sympathetic renal innervation or impairment of renal blood flow [2], pseudohyponatremia secondary to lipid and protein-rich plasma [3], excessive renal sodium excretion with SCI with associated brain injury with cerebral salt wasting [4], renal maintenance of water independent of ADH control in tetraplegic patients, coupled with a defect of intrarenal excretion of water [5], ADH induced hyponatremia caused by elevated fluid intake, and [6] disassociation of antidiuretic hormone (ADH) and the inhibitory effect of hypotonicity causing water retention despite hyponatremia [[15], [16], [17]].

With low plasma sodium, water enters brain cells causing cytotoxic cerebral edema, subsequent increase of intracranial pressure, which may end with non-cardiogenic pulmonary edema due to centrally mediated increased vascular permeability and pulmonary vasoconstriction, as observed in this patient. Neurological manifestation is usually apparent with very low levels of plasma sodium (<115 mmol/L), a condition termed hyponatremic encephalopathy with symptoms ranging from acute confusion to coma. The attempt to correct the hyponatremia was performed with hypertonic saline (NaCl 3%) infusion, fluid restriction, and loop diuretics. Sodium correction started at the rate of 250 cc/24 h (10 mmol/L/day) to avoid the risk of osmotic demyelination syndrome (ODS), which is another deadly complication that riddles the management of SCI. However, ODS can still happen following a slow correction of hyponatremia [18]. Thus, newer evidence suggests a maximum of 8 mmol/L within the 24 h up to 6 mmol/L for patients at high risk of osmotic demyelination [19]. Other evidence put more importance on the rate of diagnosis rather than the rate of treatment.

Following plasma sodium correction the patient showed no signs of clinical or neurological improvements and was pronounced dead three days afterward. Irreversible cytotoxic brain edema and pulmonary edema persisted despite the corrected sodium level. High cervical compression (C3–C4) by the pneumorrhachis might also contribute to the patient’s pulmonary complication by disrupting the sympathetic pathways and diaphragmatic function. Patients with SCI are also subjected to a multitude of pulmonary physiologic changes that include impairment of ventilator muscle performance, changes of chest wall compliance, change in ventilatory control, airflow limitation and bronchial hyper-responsiveness. Spinal shock or SCI-induced hyponatremia is less likely to be aggravated by the operation since the main purpose of decompressing the spinal cord from pneumorrhachis was achieved by the surgery. However, whether the optimal sodium correction has been mandated and the possibility of ODS cannot be ruled out. The decision on whether to operate and when to operate in cervical SCI remains to be elucidated.

## 4 Conclusion

The challenge of cervical SCI management lies in its rarity and complex pathophysiology. As demonstrated in this case report, the unexpected orientation of dural tear, extensive pneumorrhachis, hyponatremic encephalopathy and pulmonary complications all contribute to mortality. Prompt clinical stabilization, infection control, and strict biochemical monitoring and appropriate correction are mandatory. More importantly, multidisciplinary care involving pulmonology, cardiology, intensive care, nutrition, and medical rehabilitation specialists is essential to provide the best care possible for SCI patients.

## Declaration of Competing Interest

We declare no conflict of interest.

## Sources of funding

Nothing to declare.

## Ethical approval

Ethical approval exempted by our institution.

## Consent

Written informed consent was obtained from the deceased patient’s family. Information within the paper has been sufficiently anonymised not to cause harm to the patient or their family. A copy of a signed document stating this is available for review by the Editor-in-Chief of this journal on request.

## Author contribution

1 Tommy Supit: performed surgery, conception of report, data collection, data analysis, manuscript writing, revision and submission.
2 Ajid Risdianto: performed surgery, conception of report, data analysis, and manuscript revision.
3 Dody Priambada: data analysis, manuscript writing and manuscript revision.
4 Muhamad Thohar Arifin: data analysis, manuscript writing and manuscript revision.
5 Happy Kurnia Brotoarianto: performed surgery, conception of report, data collection, data analysis, and manuscript revision.

## Registration of research studies

Case report not registered.

## Guarantor

Tommy Supit and Happy Kurnia Brotoarianto.

## Provenance and peer review

Editorially reviewed, not externally peer-reviewed.
